# Public Spending on Health Services and Policy Research in Canada: A Reflection on Thakkar and Sullivan: Comment on "Public Spending on Health Service and Policy Research in Canada, the United Kingdom, and the United States: A Modest Proposal"

**DOI:** 10.15171/ijhpm.2017.113

**Published:** 2017-09-16

**Authors:** Owen Adams

**Affiliations:** Canadian Medical Association, Ottawa, ON, Canada.

**Keywords:** Health Services, Policy, Research

## Abstract

Vidhi Thakkar and Terrence Sullivan have done a careful and thought-provoking job in trying to establish comparable estimates of public spending on health services and policy research (HSPR) in Canada, the United Kingdom and the United States. Their main recommendation is a call for an international collaboration to develop common terms and categories of HSPR. This paper raises two additional questions that have an international comparative dimension: There is little doubt that public spending on HSPR represents more than the "tip of the iceberg," but how much more? And how do the countries fare on the uptake of HSPR by decision-makers? I have long speculated that probably as much or more is spent by provincial/territorial governments, regional health authorities, hospitals and other agencies on HSPR activities carried out by consultants in Canada than by the federal, provincial/territorial granting agencies. Support for this contention is provided in a paper by Penno and Gauld on spending on external consultancies by New Zealand’s District Health Boards (DHBs). Their estimate of the amount spent on consultancies in 2014/15 represents 80% of the amount spent on research by the Health Research Council of New Zealand in 2015. In terms of the uptake of research Jonathan Lomas pioneered the concept of linking researchers with decisionmakers when he became the founding Chief Executive Officer (CEO) of the Canadian Health Services Research Foundation (CHSRF) in 1997. An early assessment was promising, and it would be interesting to know if other countries have tried this. Most assessments of research uptake and impact are short-term in nature. It might be insightful to assess HSPR developments over the long term, such as prospective reimbursement through diagnosis related groups (DRGs) that has been evolving internationally for more 40+ years. In the short term the prospects for a major infusion of funding in HSPR in Canada are not promising, although there have been welcome investments in the Canadian Foundation for Healthcare Improvement (formerly CHSRF).

Vidhi Thakkar and Terrence Sullivan have done a careful and thought-provoking job in trying to establish comparable estimates of public spending on health services and policy research (HSPR) in Canada, the United Kingdom, and the United States. They have put forward two calls for action. First, they emphasize the need to assess the impact of increased investment in HSPR on health system performance and quality of care. Second, they recommend an international collaboration to develop common terms and categories of HSPR.^1^

This paper raises two additional questions that have an international comparative dimension:

- There is little doubt that public spending on HSPR represents more than the “tip of the iceberg,” but how much more? And
- How do the countries fare on the uptake of HSPR by decision-makers?

It is important to recognize that HSPR is a wide field of endeavour. The Canadian Institutes of Health Research (CIHR) defines it as “a multidisciplinary field of scientific investigation that studies how social factors, financing systems, organizational structures and processes, health technologies, and personal behaviours affect access to healthcare, the quality and cost of healthcare, and ultimately, Canadians’ health and well-being.”^2^

## How Much More Spending Is There on HSPR Than Public Spending?

As someone who has been an observer of the HSPR scene in Canada for almost 40 years, and an active peer reviewer for several HSPR granting bodies, I have long speculated that probably as much or more is spent by provincial/territorial governments, regional health authorities, hospitals and other agencies on HSPR activities carried out by consultants in Canada than by the federal, provincial/territorial granting agencies. Supporting evidence for this contention is provided in a recent paper by Penno and Gauld on spending on external consultancies by New Zealand’s District Health Boards (DHBs). In 2016 they submitted Official Information Act requests to each of New Zealand’s 20 DHBs requesting details on all external consultancies over the 3-year period from 2012/2013 to February 2016, which they subsequently refined to just those costing more than NZ$ 10 000. Thirteen DHBs agreed to provide the information, one gave them no response, four refused and two would only provide it at a significant cost. They supplemented the information with figures provided by the DHBs to the government Health Select Committee. In 2014/2015 they estimated the total expenditure at NZ$ 6 4128 553.^3^

How does this compare with public expenditure? According to the 2016 annual report of the Health Research Council of New Zealand, in 2015 research grant costs amounted to NZ$ 80 960 million; hence the consultancy expenditures represented almost 80% of the public total.^4^ To be fair this is a rough comparison. As veteran healthcare consultant Neil Stuart has pointed out, consulting expenditures on information technology and administrative systems should not be included. What would be reasonable to include as HSPR spending would be items including strategy consulting, planning, surveys, stakeholder engagement/consultation, quality improvement and evaluations (N. Stuart, personal communication, August 2017). Hunter and Frank make a similar point in their commentary, noting that the English National Health Service commissions applied HSPR from academics.^5^

It would be no easy task to identify consultancy expenditures for HSPR in Canada. In the case of hospitals, the Management Information System Standards used by the Canadian Institute for Health Information include secondary accounts for “management fees” and “professional fees not elsewhere classified.”^6^ However this would include a mixture of the items categorized by Stuart as well as items such as legal fees. In 2010 the Auditor General of Ontario released a special report on the use of consultants in selected organizations. It contained three recommendations intended to improve the reporting on the procurement and management processes with consultant use generally.^7^ Suffice it to say that few would argue that public non-granting agency spending on HSPR activities is trivial, and moreover one might venture that fewer results come into the public domain as compared to the output of public agency-funded research.

Another important category of public spending on HSPR activities is that of research funded by commissions and task forces, which have been very popular in Canada. A key example is the extensive external research program of the 2001-2002 Royal Commission on the Future of Health Care in Canada, led by Roy Romanow. It commissioned 40 discussion papers from experts from across Canada and internationally, as well as 3 major research projects.^8^

## How Do Countries Fare on the Uptake of HSPR?

In 1997 McMaster University professor Jonathan Lomas produced an influential working paper that examined the challenges and prospects of moving research into decision-making in the health sector, which he referred to as getting “beyond the sound of one hand clapping.”^9^ Lomas suggested that a key reason for the lack of uptake of research was that researchers and decision-makers were working independently of each other; “it is like two people trying to assemble a jigsaw puzzle, each with half of the pieces…but each working in a separate room.”^9^ He added that a further problem was that both researchers and decision-makers failed to recognize that research and decision-making are not discrete events, but rather iterative processes, and he proposed that both parties should have more opportunities to engage in exchange throughout their processes. Another major contribution of the paper was that Lomas developed a categorization of the different types of decision-maker, including clinical, legislative, administrative and industry decision-makers.

Lomas had an opportunity to put these ideas into action when he became the founding Chief Executive Officer (CEO) of the Canadian Health Services Research Foundation (CHSRF). The CHSRF was established through an initial endowment of $65 million in the 1996 federal budget. As described in the budget, the purpose of the fund was to “bring together partners from provincial governments, health institutions and the private sector…the research will be practical in nature…By jointly setting priorities and pooling efforts, the results of the research should be more readily and widely adopted to the benefit of all Canadians.”^10^

Lomas introduced two innovations in the CHSRF granting process. First, all researchers were required to have a decision-maker partner. Second, grant proposals were adjudicated based on an equal weighting of scientific merit and potential impact, and the review panels comprised researchers and decision-makers representing the different types. An early evaluation of the CHSRF experience by Ross et al reported that on balance, both researchers and decision-makers felt it was a beneficial experience. The researchers reported benefits including a greater focus on application, access to data sources and a greater understanding of the decision-making environment. Decision-makers reported that their involvement had made them more reflective about their activities.^11^ It would be interesting to know how successful similar initiatives in the United States and United Kingdom have been in improving the relevance and uptake of HSPR. The United States, for example has Academy Health, which was established in 2000 and acts as a broker of information between health services researchers, policy decision-makers and practitioners.^12^

The CIHR has just launched a new program of Health System Impact Fellowships that will embed post-doctoral Fellows in health service organizations and it will be interesting what impact this has on both researchers and decision-makers.^13^

With regard to Thakkar and Sullivan’s proposed international collaboration, while this is certainly a good idea, I would suggest that “charity begins at home.” In 1998, Lewis and colleagues put forward an idea for an “evidence-based decision-making trade show” that would bring together governors, providers, managers, policy-makers and industry with the community of healthcare researchers in academic and non-academic settings from across Canada. The authors postulated that this would offer several advantages such as permitting a state-of the-art exchange of needs, products, strategies, replicable successes and pitfalls to avoid.^14^ Such a trade show has yet to occur. Researchers, clinicians and healthcare administrators largely attend their own conferences, and probably mainly at the provincial level.

Returning to the concept of international collaboration this is something where the consulting industry could help. The large consulting firms operate in numerous countries and one would imagine that they share methods, findings and key lessons across them. For example, KPMG’s Mark Britnell developed a comparative 25-country study *In Search of the Perfect Health System* based on his experience of working in 60 countries. He concluded that there is no perfect health system, but if there was, it might be based on 12 qualities from the best country experience for each, such as the values and universal coverage of the UK and the research and development of the United States.^15^

## Prospects for a Big Boost in Funding for HSPR

In their conclusion Thakkar and Sullivan suggest that it might be useful to set a target for HSPR funding, as was done in England in the 1990s. Tamblyn et al have suggested that whereas knowledge-intensive industries invest 5% (of their budgets) in research and development an appropriate investment in HSPR for Canada in 2014 would have been $11 billion (based on total health spending of $215 billion).^16^ Considering that CIHR’s total research budget is roughly $1 billion this would clearly be a tall order.^17^ One of the challenges in securing funding for health research in general and HSPR specifically is the need to demonstrate return-on-investment. An added challenge continues to be the issue identified by Lomas in 1997 of the failure to appreciate research and decision-making as a process rather than as a discrete event. This results in a short-term focus on evaluation thus making it difficult to demonstrate significant impacts. In this regard it might be helpful to cultivate a longer-term view of the impact of HSPR. A case in point would be what I would consider to be the most successful HSPR story ever, and that is the adoption of prospective reimbursement by diagnosis related groups (DRGs) that began with research conducted by Fetter and Thompson at Yale University in the 1970s.^18^ DRGs were initially designed as a means of monitoring utilization of resources and quality of care, but were adopted by the State of New Jersey in 1978 as a prospective payment mechanism and nationally by the US Medicare program in 1983.^19^ DRGs are now widely used internationally,^20^ and the Centers for Medicare and Medicaid Services is extending the concept further with “bundled payments”; prospective payment that cover all health services for an episode of care, including hospital care, physician care home care and rehabilitation.^21^ Canada developed its own version of DRGs – Case Mix Groups in the 1980s but it is only in the last decade that some provinces have started to adopt them as a funding tool.^22^ DRGs are no panacea to be sure but they are an excellent case study of the uptake of research.

In the short-to-medium term the prospects of a major infusion of resources into HSPR do not appear promising. In 2015 the Advisory Panel on Healthcare Innovation, chaired by David Naylor, recommended a *Healthcare Innovation Fund* that would ramp up to $1 billion annually by 2020 that would support initiatives related to HSPR^23^ but to date there has been no major commitment by the federal government, aside from new investments in the Canadian Foundation for Healthcare Improvement (formerly CHSRF) (CFHI) totaling $90 million in the 2016 and 2017 federal budgets plus ongoing annual funding of $17 million.^24^ CFHI has been doing unique work in Canada to promote the spread and scale-up of innovative models of care. In 2014-2015 it funded 19 teams across Canada to implement the INSPIRED care model for chronic obstructive pulmonary disease and is now in the competitive process of scaling up some of these sites to six health regions or jurisdictions (eg, province).^25^

Most recently the report of Canada’s Fundamental Science Review, also chaired by Naylor, has recommended an investment of $485 million over four years to fund investigator-led research, although this would be allocated across the federal granting councils.^26^ Science Minister Kirsty Duncan welcomed the report’s April, 2017 release, but it will remain to be seen in the 2018 federal budget if it will result in a significant investment.^27^

## Conclusion

In conclusion – there is little comfort in the July, 2017 release of the Commonwealth Fund’s 11-country comparative study. The findings show that Canada has traded places with France to move up to occupy ninth place; on the five individual dimensions that make up the composite score, Canada ranks no better than sixth.^28^ There seems little doubt that greater investment in HSPR would help the situation, but linkages need to be stronger between researchers and decision-makers, and there is a need for greater sharing within Canada and internationally.

## Ethical issues

Not applicable.

## Competing interests

Author declares that he has no competing interests.

## Author’s contribution

OA is the single author of the paper.
